# Frog size on continental islands of the coast of Rio de Janeiro and the generality of the Island Rule

**DOI:** 10.1371/journal.pone.0190153

**Published:** 2018-01-11

**Authors:** Raoni Rebouças, Hélio Ricardo da Silva, Mirco Solé

**Affiliations:** 1 Programa de Pós-Graduação em Ciências Biológicas–Biologia Animal, Universidade Federal do Espírito Santo. Avenida Fernando Ferrari, Vitória, Espírito Santo, Brazil; 2 Laboratório de Herpetologia, Universidade Federal Rural do Rio de Janeiro. Caixa Postal, Seropédica–Rio de Janeiro, Brazil; 3 Departamento de Ciências Biológicas, Universidade Estadual de Santa Cruz. Rodovia Jorge Amado, Salobrinho, Ilhéus–Bahia, Brazil; University of Sao Paulo, BRAZIL

## Abstract

Island Rule postulated that individuals on islands tend to dwarfism when individuals from mainland populations are large and to gigantism when mainland populations present small individuals. There has been much discussion about this rule, but only few studies were carried out aiming to reveal this pattern for anurans. Our study focused on measuring the size of individuals on islands and to find a possible pattern of size modification for insular anurans. Individuals were collected on continental islands, measured and compared to mainland populations. We selected four species with different natural history aspects during these analyses. Island parameters were compared to size of individuals in order to find an explanation to size modification. Three of the four species presented size shifting on islands. *Ololygon trapicheiroi* and *Adenomera marmorata* showed dwarfism, *Boana albomarginata* showed gigantism and in *Thoropa miliaris* there was no evident size modification. Allometric analysis also revealed differential modification, which might be a result of different selective pressures on islands in respect of mainland populations. Regression model explained most of the size modification in *B*. *albomarginata*, but not for the other species. Our results indicate that previous assumptions, usually proposed for mammals from older islands, do not fit to the anurans studied here. We support the assumption that size modification on islands are population-specific. Hence, in *B*. *albomarginata* some factor associated to competition, living area and isolation time might likely be responsible for gigantism on islands.

## Introduction

Effects of isolation on the size of animals inhabiting islands have attracted attention of many researchers [1–5]. The first observations describing size differences in individuals from islands were made by Foster [6] for mammals. Posteriorly, Van Valen [7] presented a generalization of these observations as a rule, that became known as “Island Rule”. In general, this rule describes a gradual tendency of individuals from island populations towards gigantism when the continental forms (the source population) are formed by relatively small individuals and towards dwarfism when mainland individuals are relatively large.

Much is still discussed on the causes of continent-island size variation [2, 3, 8], and although many studies have referred to the Island Rule, applying it to several groups [9, 10, 11], just a few studies have focused on amphibian populations [12–14]. Perhaps because such a rule was developed with long distance dispersal and volcanic islands in mind, and the effects described being related to species in the continent being considered the ancestral stock for those on islands [1, 2, 15], amphibians were excluded from such considerations because these animals do not occur on those islands. The only study that considered anuran amphibians in the analyses adapted the rule to continental islands, which were formed at the end of the Pleistocene as a result of sea level transgression [16].

Anura comprises more than 7600 described species in 56 families [17], and this order is the most diverse group of terrestrial vertebrates on the planet. On islands, anurans are particularly interesting because: (i) for most of the species, dispersal across the ocean is impossible, or highly improbable, consequently most species are isolated; (ii) anurans are ectotherms, with different energetic needs, behavioral patterns and habitat use in relation to mammals; (iii) such animals present diverse repertoire of reproductive modes [18]; and (iv) anurans are relatively easy to survey [19].

Earlier studies designed to investigate differences in size between island and nearby continental populations of amphibians included a bromeligenous species [14], pond users [20] and habitat generalists [12, 13]. However, in any of these studies the natural history of the species was explored aiming to explain factors related to the causes of size differences as proposed by Palkovacs [21] and Lomolino et al. [8].

In the present study, we took advantage of a feature of the Brazilian coast, which presents hundreds of coastal, or land-bridge islands, which are fragments formed by sea level transgression by the end of the Pleistocene [21]. In terms of habitat, most of Brazilian coastal islands are within the Atlantic Rainforest domain, and have rich anuran communities [22, 23], including some endemisms [22, 24, 25]. Islands on the southern coastal shore of Rio de Janeiro, locally known as *Costa Verde*, are located within a protected area that is recognized and named as two bays: *Baía da Ilha Grande* and *Baía de Sepetiba*. These bays harbor hundreds of islands of different magnitudes and some of which have already been studied and data regarding anuran surveying have also been published [22, 23, 26]. Because the age and constitution of these islands, we designed this study in order to: (i) document and compare the size of individuals belonging to continental and insular populations relative to four different species; (ii) investigate the relationship between size of individuals and measurable features of the islands; and (iii) compare observed size differences for each species investigated with results from other studies, to investigate the existence of a general pattern that explains anuran dimorphism between coastal islands and mainland.

## Materials and methods

### Samples and study area

**Fig 1:**
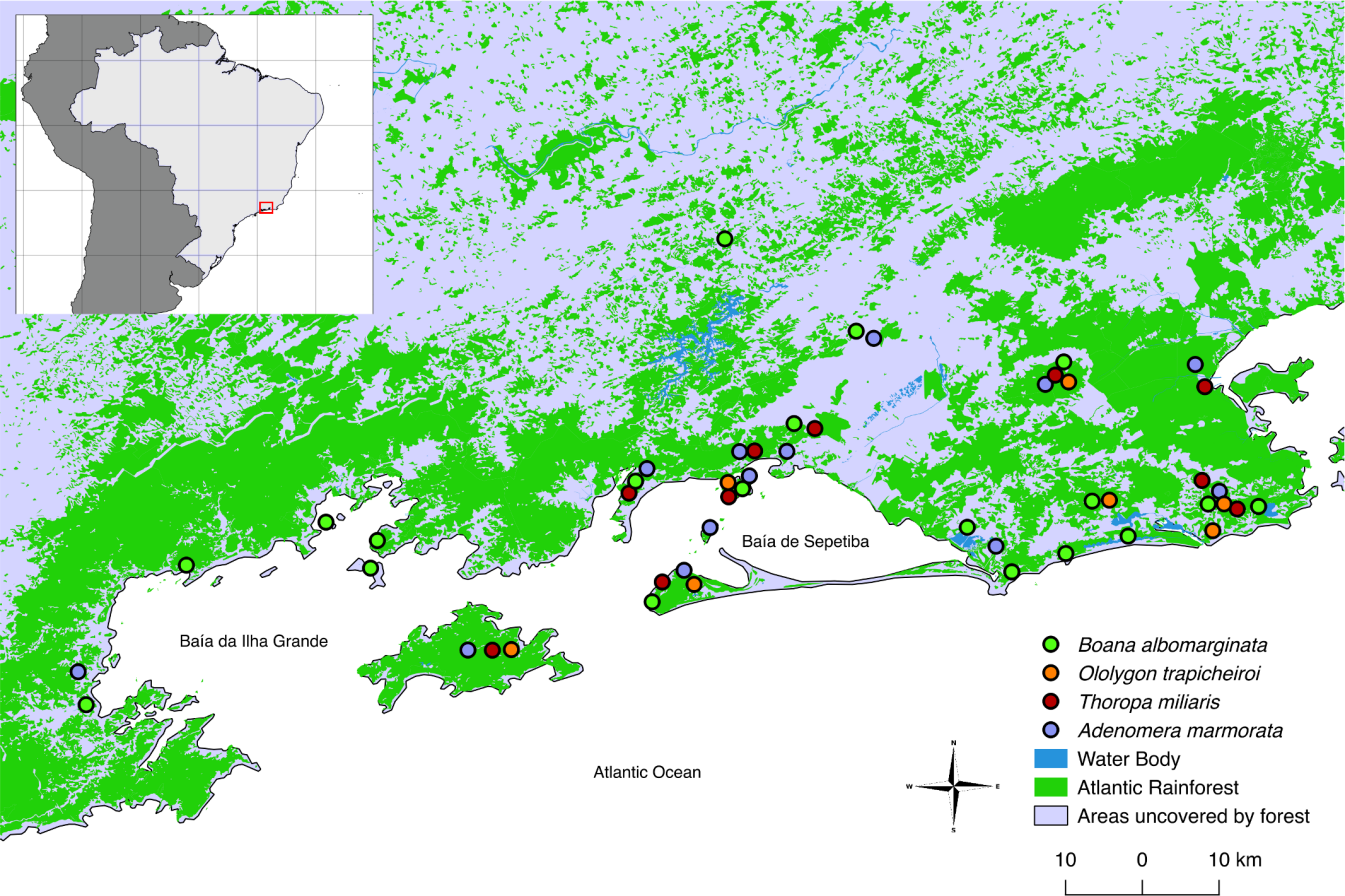
Collecting sites. Distribution of populations sampled in this study in *Costa Verde*, state of Rio de Janeiro.

Specimens were sampled along the *Costa Verde*, state of Rio de Janeiro, Brazil, between the municipalities of Rio de Janeiro (22°54’13”S, 43°12’35”W) and Paraty (23°13’0”S, 44^a^43’4”W), and on nearby islands, that are: Ilha de Itacuruçá (22°56’29”S, 43°53’26”W), Ilha de Jaguanum (23°0’6”S, 43°55’38”W) and Ilha da Marambaia (23°3'39"S, 43°47'19"W), in *Baía de Sepetiba*, and Ilha Grande (23°8'26"S, 44°14'50"W), Ilha da Gipóia (23°2’35"S, 44°21’40”W) and Ilha de Itanhangá (22°59'22"S 44°24'46"W), in *Baía da Ilha Grande* (Fig 1).

These islands were chosen by presenting different characteristics from each other concerning relief, area, and anuran diversity [22]. Data regarding anuran species richness by locality used here were presented by Bittencourt-Silva and Silva [22] and those relating to the bay topography based on nautical charts produced by the Brazilian Navy [27]. Mainland areas were considered as one locality during the analyses, assuming some degree of connectivity between these populations, as already demonstrated for rodents of the genus *Nectomys* [28].

**Fig 2:**
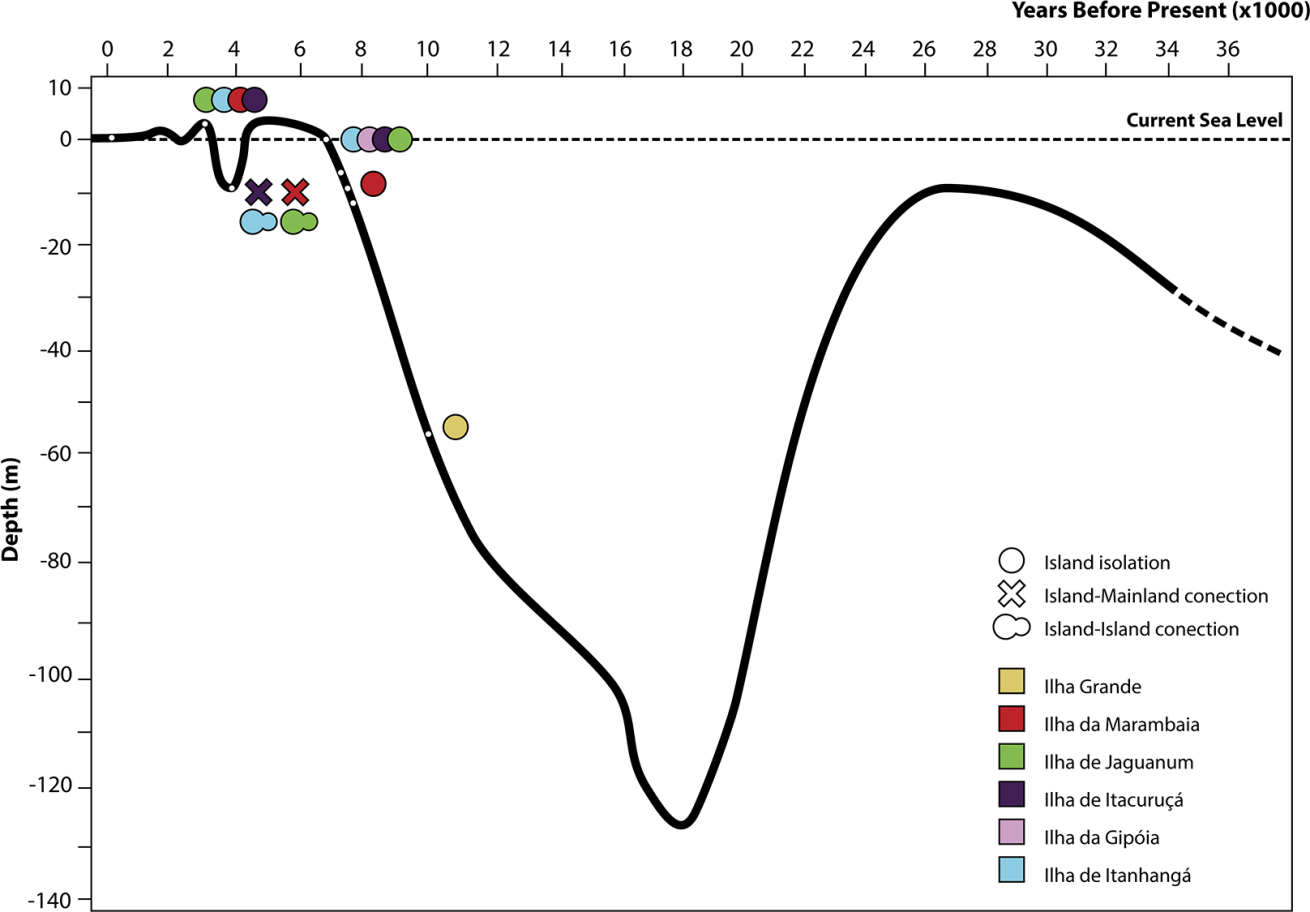
Holocenic sea level. Sea level variation from 36 kybp to present days (depth confidence interval: ± 1 m, standard deviation of time: ± 280 years) (according to the Brazilian Navy [27] and adapted from Suguio [32]).

The islands of the archipelago where the study was conducted are land-bridge islands (or continental islands), formed by flooding process of the continental platform as a result of oceanic transgression [16, 29]. Palaeogeographic evolution and consequently island formation might be divided in 5 stages: (i) Before the end of the last glacial maximum (~ 18 thousand years before present, or kybp) the average sea level was nearly 120 m below the current conditions [30] and the continental platform was exposed and likely covered by Atlantic Rainforest vegetation [31]. (ii) At the end of the last glacial maximum, between 18 and 4 kybp, as a consequence of global warming, sea level rose resulting on flooding of the coastal lowlands (marine transgression) and only the areas above the maximum sea elevation, represented by some coastal hills, remained exposed, forming the coastal islands currently observed [30]. (iii) Approximately between 4.5 and 3.5 kybp a new regression occurred reducing the sea level from 3 m above to 10 m below the current level. (iv) Between 3.5 and 3 kybp those connections were lost due to a new marine transgression, rising sea level from 10 m below to 3 m above the current level. (v) After this last transgression at 3 kybp, a regression dropped the sea level to the conditions observed today, and, despite a small oscillation at 2 kybp, Ilha de Itacuruçá, Ilha de Itanhangá and Ilha da Jaguanum remained isolated from nearby islands. Ilha da Marambaia remains connected to the mainland by 100 m of sand strip at the narrower point [26], but not sufficient to keep connectivity between anuran populations due to the absence of reproduction habitats [23] (Fig 2).

### Species investigated for size variation

Four species were sampled during the morphometric analyses: *Thoropa miliaris* (Spix, 1824), Cycloramphidae, a forest species that reproduces in films of water, not necessarily, but sometimes associated to waterfalls. The reproduction of this species involves parental care, territorial contests between males and pronounced territorial protection [33]. *Adenomera marmorata* Steindachner 1867, Leptodactylidae, a ground-dweller forest species with reproduction and feeding restricted do leaf litter [34, 35]. *Boana albomarginata* (Spix, 1824), Hylidae, a lowland species that reproduces in ponds in open areas and forest borders [36] and in which males present aggressive territorial protection [37]. Finally, *Ololygon trapicheiroi* (A. Lutz and B. Lutz, 1954), Hylidae, typical of forest with reproductive sites associated to ponds at the margin of small streams and creeks [38]. These species were chosen based on information that could guarantee the usage of different habitats, life histories, and that previous surveys indicated that they were abundant in at least three islands in the study area [22] and in nearby mainland areas (a list of the material examined is in S2 Table). All individuals used in this study are deposited in the Coleção do Laboratório de Herpetologia da Universidade Federal Rural do Rio de Janeiro and in the Coleção de Herpetologia do Museu Nacional do Rio de Janeiro (acronyms RU and MNRJ, respectively).

### Measurements

Individuals were measured with digital calipers to the nearest of 0.01 mm. The following measurements were taken: snout-vent length (SVL), head length (HL), head width (HW), inter-nostril distance (IND), inter-orbital distance (IOD), eye-nostril distance (END), eye diameter (ED), tympanum diameter (TD), femur length (FL), leg length (LL), tarsus length (TL) and foot length (FTL). In order to eliminate possible biases caused by immature specimens in the sample, we surveyed only adult individuals. Such a strategy was guaranteed with observation of secondary sexual characters, such as nuptial spines in *T*. *miliaris* males [39], nuptial pads in males of *O*. *trapicheiroi* [40], expanded vocal sacs in males of *B*. *albomarginata*, and differentiated rostral skin in males of *A*. *marmorata*.

### Analyses

Normality of data distribution was tested through the Anderson-Darling normality test in the case of multivariate analysis and Shapiro-Wilk normality test in the case of univariate analysis. Data were log_10_ transformed when normality premises were not accomplished. In order to evaluate general size difference of anurans between each island relative to mainland populations, we used Student's t-test using SVL of individuals. To verify allometric differences between frogs from each island and mainland populations we used a Principal Components Analysis (PCA), and the difference was calculated with one-way ANOVA based on the first PCA component when cumulative variance percent was significantly high. Moreover, we applied the Hotelling’s T^2^ test using components from PCA which together accomplish a high cumulative variance percent.

In order to evaluate possible causes of size differences in populations that are significantly distinct on islands, a multiple regression analysis was carried out between SVL and intrinsic characteristics of the islands. Islands’ characteristics evaluated were (i) anuran species richness for each island (*S*) (for the diversity in each island we used data presented by Bittencourt-Silva and Silva [22] as an indirect way to evaluate inter-specific competition), (ii) the lowest distance between one island and the continent, obtained through Google Earth [41]; and (iii) as an indirect way to evaluate degree of isolation, and island surface, we calculated an approximation of conic area formulae:

$$S_{island}=\pi R(g+R)$$

Where *S*_*island*_ = island surface; *R* = radius of base, considering island flat area as a circular base; and *e* = radius of circular sector, or the distance between perimeter and the highest point of the island. Island surface here was used as an indirect way to evaluate amount of habitat putatively available, since in larger areas probably there is more habitat for a certain species [8, 42]. All analyses were performed in R 3.3.0 [43] with confidence interval of 95%.

## Results

Seven hundred individuals were measured of all four species from islands and from 37 localities that composed the mainland. The sample size for each species and from each locality is in Table 1.

**Table 1:** Size per locality. Mean of SVL for each species by locality and number of examined specimens.

Species	MLD	ITA	JAG	MAR	GRD	GIP	ITN
*Thoropa miliaris*	57.2 ± 8.6 (*n* = 34)	53.8 ± 6.8 (*n* = 19)	–	56.2 ± 7.2 (*n* = 32)	53.6 ± 7.9 (*n* = 26)	–	–
*Adenomera marmorata*	20.5 ± 1.3 (*n* = 99)	19.6 ± 1.3 (*n* = 19)	19.6 ± 1.8 (*n* = 14)	19.1 ± 1.8 (*n* = 25)	20.4 ± 1 (*n* = 26)	–	
*Boana albomarginata*	43.9 ± 2.8 (*n* = 108)	46.8 ± 1.9 (*n* = 34)	–	45.4 ± 0.8 (*n* = 33)	–	51.9 ± 1.5 (*n* = 12)	–
*Ololygon trapicheiroi*	26.2 ± 1.1 (*n* = 110)	26.5 ± 0.7 (*n* = 15)	–	25.9 ± 0.7 (*n* = 43)	25.1 ± 0.9 (*n* = 35)	–	
							(*n* = 14)

MLD, Mainland populations; ITA, Ilha de Itacuruçá; JAG, Ilha de Jaguanum; MAR, Ilha da Marambaia; GRD, Ilha Grande; GIP, Ilha da Gipóia; ITN, Ilha de Itanhangá, *n*, specimens number.

**Fig 3:**
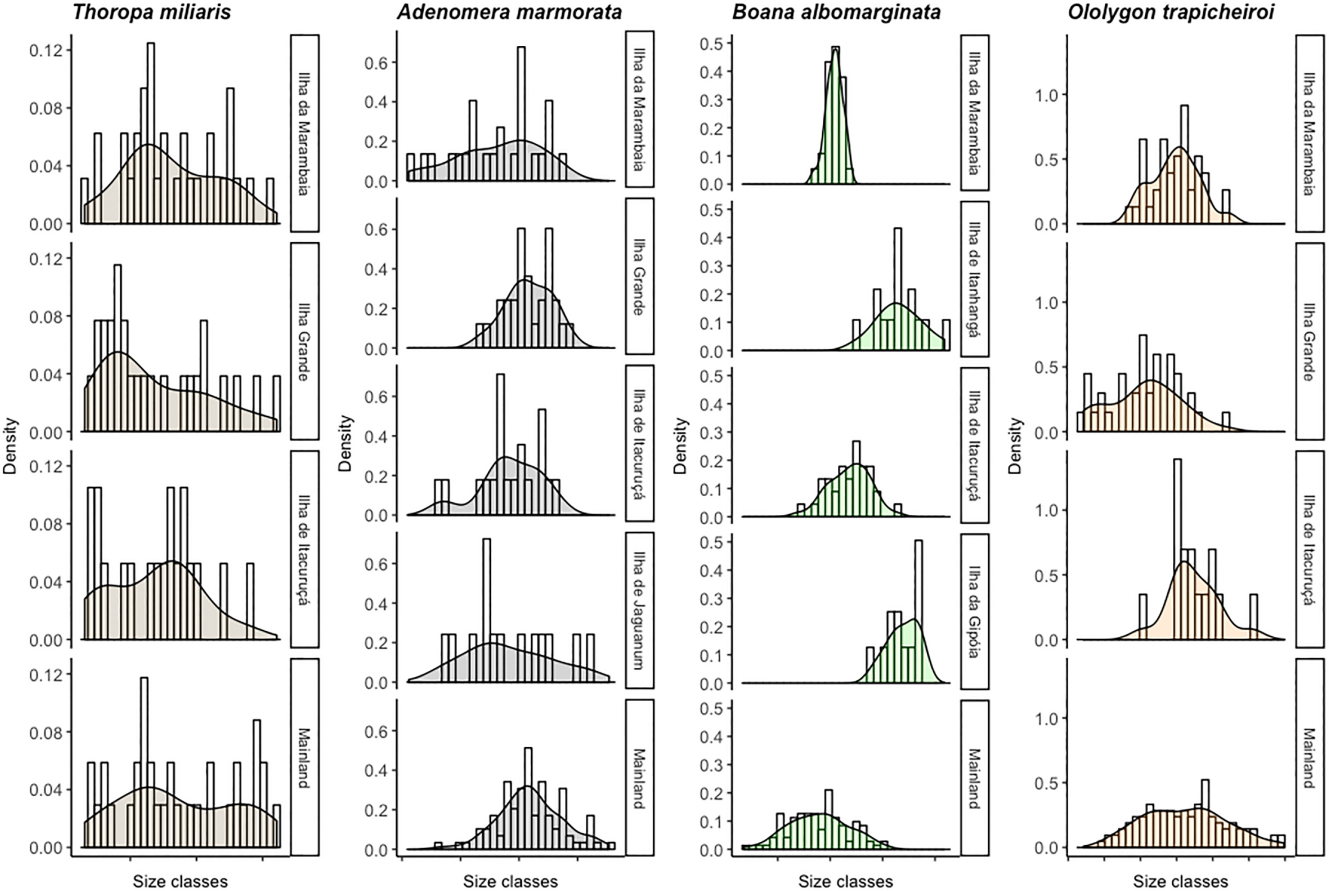
Size shifting. Distribution of individuals in each size category by sampled locals.

**Fig 4:**
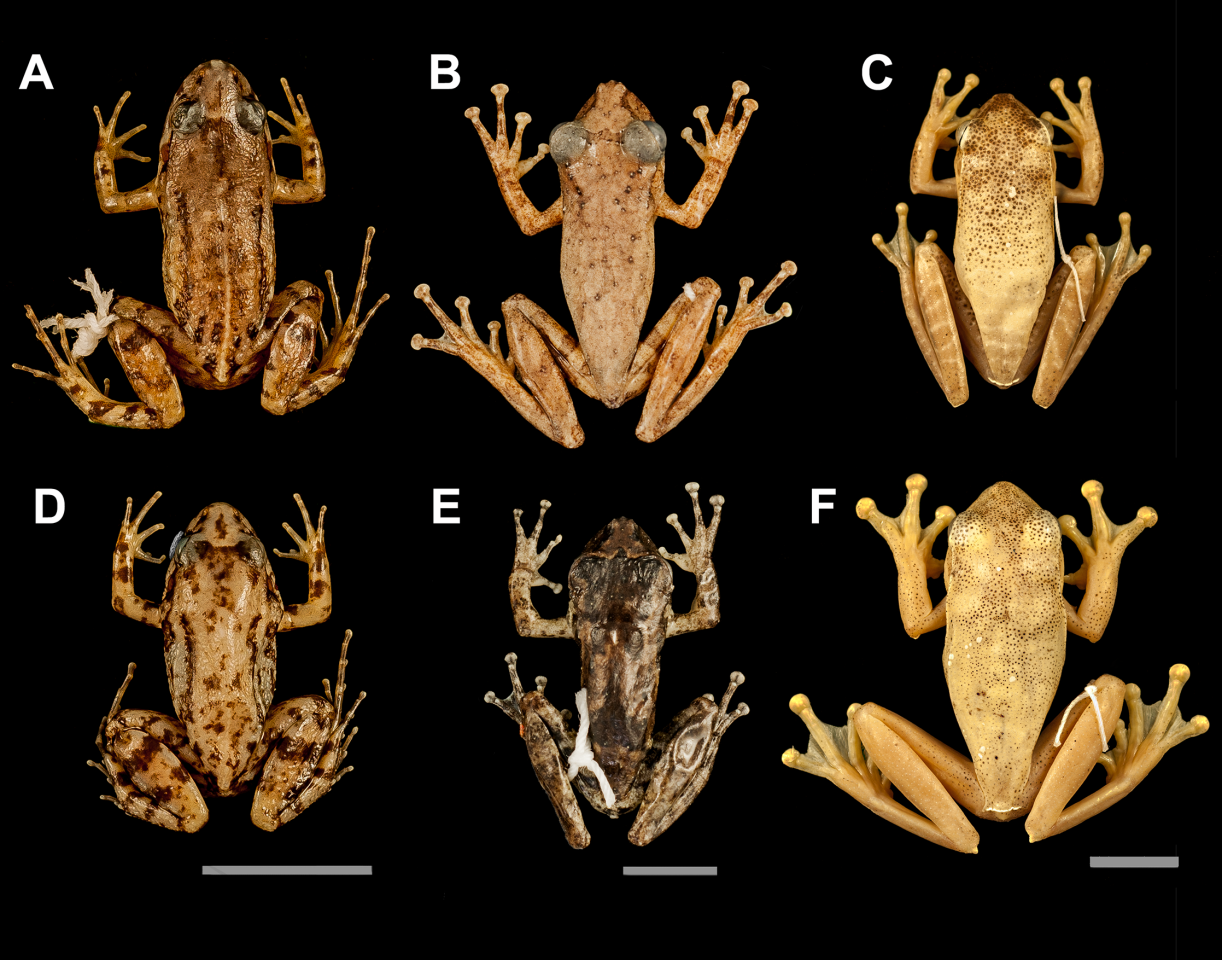
Island versus mainland populations. Species that presented significant size difference in islands: *Adenomera marmorata* (A: Mainland and D: Ilha da Marambaia), *Ololygon trapicheiroi* (B: Mainland and E: Ilha Grande) and *Boana albomarginata* (C: Mainland and F: Ilha de Itanhangá) (scale: 1 cm).

Student's t-test showed that there was no difference for *T*. *miliaris* between mainland and Ilha da Marambaia (t = 0.55, p = 0.59), Ilha de Itacuruçá (t = -1.61, p = 0.11) and Ilha Grande (t = -1.64, p = 0.11). Yet, for *A*. *marmorata*, there was significant difference between mainland and Ilha da Marambaia (y = -3.61, p = 0.01) and Ilha de Itacuruçá (t = -2.6, p = 0.01), but such difference was not observed for Ilha de Jaguanum (t = -1.74, p = 0.1) and Ilha Grande (t = 0.26, p = 0.79). In *B*. *albomarginata* there was no difference between individuals from mainland and Ilha de Itacuruçá (t = 1.61, p = 0.12), however, there was difference between mainland and Ilha da Marambaia (t = -2.25, p = 0.03), Ilha da Gipóia (t = 11.24, p<0.01) and Ilha de Itanhangá (t = 8.33, p<0.01). In *O*. *trapicheiroi* there was no difference between individuals from mainland and Ilha da Marambaia (t = -1.76, p = 0.08) and Ilha de Itacuruçá (t = 1.15, p = 0.26), however, there was difference between individuals from mainland and Ilha Grande (t = -5.63, p<0.01) (Figs 3 and 4)

**Fig 5:**
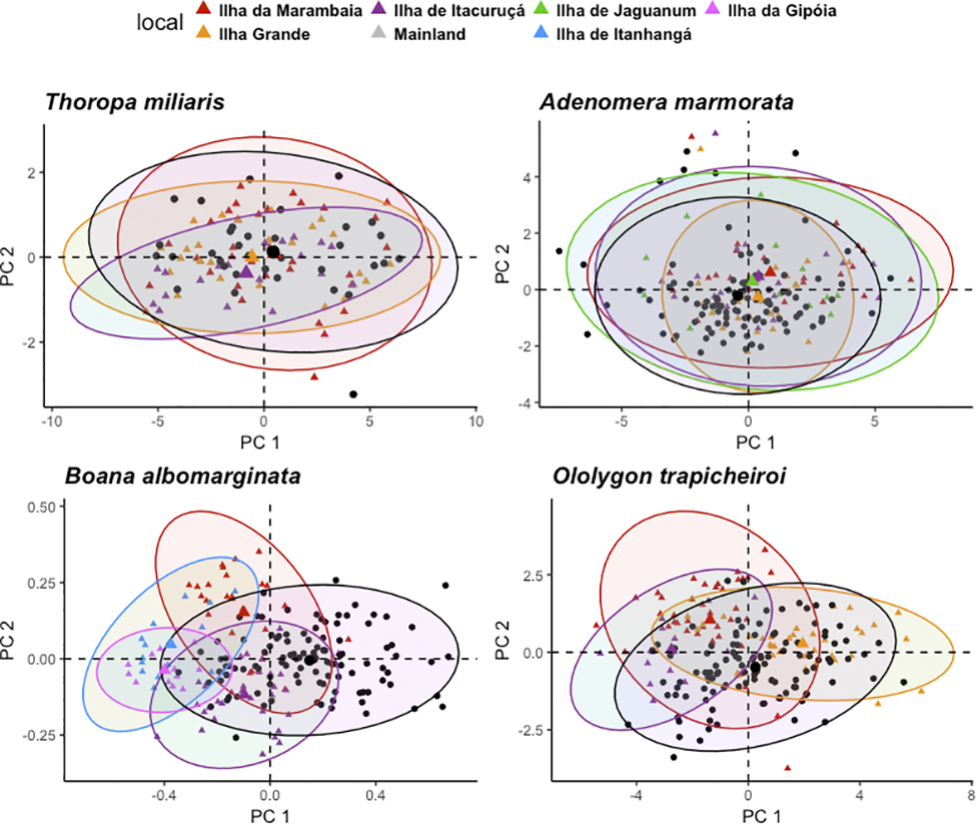
Allometric shifting. Principal Components Analysis (PCA) considering each island and mainland populations for each species.

Principal Components Analysis showed that the percent of variation in *T*. *miliaris* relative to the first component was almost 80%, and to reach this level in *A*. *marmorata* and *O*. *trapicheiroi* it was necessary to consider four components (79.19% and 79.11%, respectively). For *B*. *albomarginata* we had to consider accumulated variation of three components to reach 75.71% (S1 Table). In *T*. *miliaris* there is a more evident overlap between measurement sets, with the set of Ilha de Itacuruçá being more different than the other sites, while in *B*. *albomarginata* there was less overlap between islands and mainland populations (Fig 5).

ANOVA revealed that there was no allometric difference between localities for *T*. *miliaris* (F = 1.1, p = 0.35). Hotelling’s T^2^ test showed that for *A*. *marmorata* there was a significant allometric difference between mainland and Ilha da Marambaia (T^2^ = 7.52, p = 0.01) and Ilha de Itacuruçá (T^2^ = 3.68, p = 0.01), however there was no difference for individuals from Ilha Grande (T^2^ = 1.92, p = 0.13) and Ilha de Jaguanum (T^2^ = 0.77, p = 0.51). For *B*. *albomarginata* there was significant difference between individuals from mainland and Ilha da Marambaia (T^2^ = 28.88, p<0.01), Ilha de Itacuruçá (T^2^ = 24.46, p<0.01), Ilha de Itanhangá (T^2^ = 26.8, p<0.01) and Ilha da Gipóia (T^2^ = 26.12, p<0.01). For *O*. *trapicheiroi* there was significant difference between individuals from mainland and Ilha da Marambaia (T^2^ = 12.72, p<0.01), Ilha de Itacuruçá (T^2^ = 20.83, p<0.01) and Ilha Grande (T^2^ = 2.79, p = 0.04).

Through a multiple regression analysis it was possible to evaluate that for *B*. *albomarginata* the resultant model explains most of the size variation on the islands, while for *A*. *marmorata* and *O*. *trapicheiroi* the explaining factor of regression is very low. For *T*. *miliaris*, the regression model was not performed because the low size variation of this species was not correlated to any of the island variables (Table 2).

**Table 2:** Multiple regression. Results of multiple regression in all species using factors of islands.

Species	S_island_	S	Dist	*p*	R^2^
*Thoropa miliaris*	0.16	0.21	–	–	–
*Adenomera marmorata*	0.04*	0.31	0.48	0.02*	0.11
*Boana albomarginata*	<0.001*	<0.001*	0.01*	<0.001*	0.72
*Ololygon trapicheiroi*	<0.01*	0.21	–	<0.001*	0.29

S_island_, calculated surface of islands; S, richness of anuran species on islands; Dist, island-mainland distance in kilometers; *p*, p-value of F statistics; R^2^, explaining factor of multiple regression.

* significant results.

## Discussion

Meiri et al. [3], in a study analyzing size of island mammals, did not find any support to island rule predictions when considering phylogenetic relationship to island-mainland comparison, concluding that the patterns observed by Foster [6] probably are clade-specific, and not size-specific. Even though some mammal species present dwarfism and gigantism in island populations in relation to closely related mainland species [1, 2, 6], in other groups as turtles [44], birds and lizards [45] size shifting patterns are differentiated, with dwarfism in smaller bodied species, gigantism in larger bodied species and species with no size shifting. Our results, although considering much younger islands than most of those considered by Lomolino [1, 2], Meiri et al. [3] and Foster [6], indicated that size of individuals in island populations is different among islands and mainland populations, since three of four species showed differences in size.

Except for *Thoropa miliaris*, we observed intra-specific variation (i. e. dwarfism or gigantism) in island-mainland comparisons. This result suggests that size shifting in island populations is a population-specific effect. Similarly to *Rhinella ornata* [12], island populations of *Ololygon trapicheiroi* and *Adenomera marmorata* presented dwarfism in some islands, and three of four island populations of *B*. *albomarginata* presented individuals larger than mainland populations, such as *Phyllodytes luteolus* [14]. On the other hand, island populations of *Thoropa miliaris* did not show any size modification in relation to mainland ones, and *Fejervarya limnocharis* [20] presented, depending on the island, gigantism and dwarfism.

Size modifications observed here, added to variation reported in other anuran species [12–14, 20], indicate that the island rule *sensu* Lomolino [1, 2] is not a convincing model to explain island-mainland body variation in anurans. *B*. *albomarginata*, a medium-sized species, is significantly larger in three of four islands, *T*. *miliaris*, also a medium-sized species but larger than *B*. *albomarginata*, presented no size difference, and the other two smaller species, *O*. *trapicheiroi* and *A*. *marmorata*, presented dwarfism in one of three islands and in two of four islands, respectively (Fig 3) (Table 1). Montesinos [12] reported dwarfism in *R*. *ornata* (average SVL of 65 mm in mainland) in two of three islands, Mageski et al. [14] reported gigantism in *Phyllodytes luteolus* (average SVL of 21 mm in mainland), and Wu et al. [20] described for *Fejervarya limnocharis* (average weight of 5 g in mainland) both dwarfism and gigantism in islands. Therefore, our results indicate that this pattern must not be generalized for all island vertebrates [44], as attempted by Lomolino [1]. Gigantism or dwarfism probably are not dependent of co-specifics or closely related species from mainland [45]. Moreover, size shifting observed in isolated species from islands may be due to characteristics of species and their relationship with island environment, in other words, of effects resulting of natural selection process, such as reduction of predation pressure, competition and resources limitation [3, 8].

Besides total size difference, Hotteling’s T^2^ test revealed allometric difference in *O*. *trapicheiroi*, *B*. *albomarginata* and *A*. *marmorata*. However, these differences were not indicated for a similar treatment using ANOVA for *T*. *miliaris*. Such a result was expected since we did not verify size difference on islands in relation to mainland populations in this latter species. These allometric patterns might be a result of idiosyncratic selective pressures on islands in comparison to mainland over time. Although the time may be, at first, considered short for such effects to be observed, examples from Australian snakes’ changes in body proportion may indicate otherwise [46]. Samples of older collection and freshly collected specimens of the snakes *Pseudechis porphyriacus* and *Dendrelaphis punctatus* indicated that the skull suffered reduction while the body diameter increased, in a period of less than 100 years as a result of exposure to the introduced South American toad *R*. *marina*, that acted as a putative selection agent [46].

Multiple regression models indicated that, among insular populations that showed dwarfism/gigantism, island-describing variables influenced size differently. Island surface was the only variable correlated with size shift in populations of *A*. *marmorata* and *O*. *trapicheiroi*. For populations of *B*. *albomarginata*, however, all variables investigated showed correlation with size. In *A*. *marmorata* and *O*. *trapicheiroi* multiple regression models showed that island surface, anuran richness on island and island/mainland distance were responsible for 11% and 29% of size variation, respectively. Such a finding reveals that despite the influence of area, other factors must be responsible for dwarfism on islands, and ulterior studies are necessary to verify the influence of other variables related to dwarfism. However, for *B*. *albomarginata* these factors explained most of size variation on islands (72%), meaning that, in this species, intrinsic characteristics related to competition, living area and isolation time must be critical on gigantism. As reported in other studies, males of *B*. *albomarginata* present high investment in territorial protection and aggressive interaction, and large females tend to choose larger males for mating [37, 47]. If associated to sea level oscillation (Fig 2) and reduction of suitable habitats to reproduction [26], such adaptions could favor prevalence of larger individuals in island populations. However, these behavioral characters were not quantified here and further studies concerning advertisement call, which is strongly related to body mass in several species [13, 48], and mate success in island/mainland comparison should evaluate these questions.

There is a need for more studies that aim to evaluate other possible factors that may explain size shifting in island populations, as abundance [14, 20], microhabitat availability, among others. We defend that dwarfism/gigantism cause-effect relation may be refined. We present here evidences that dwarfism and gigantism effects on islands, at least for anurans, may be population-specific, and not clade-specific as proposed by Meiri et al. [45] and Itescu et al. [44], or size-specific, as proposed by Lomolino [1, 2], Foster [6] and Van Valen [7]. Therefore, we maintain the proposed by Lomolino et al. [8], that insularity effects observed in body size depend on ecological specific characteristics.

## Supporting information

S1 Table Examined material. List of specimens examined available in the Laboratório de Herpetologia da Universidade Federal Rural do Rio de Janeiro and in the Coleção de Herpetologia do Museu Nacional do Rio de Janeiro.
(DOCX)

S2 Table PCA summary. Loadings of the first five principal componentes of the PCA with proportion of variance (PoV—%) and cumulative proportion of variance (CpoV—%).
(DOCX)
