# Supplementary material for: Frog size on continental islands of the coast of Rio de Janeiro and the generality of the Island Rule
Source: PLoS One. 2018 Jan 11;13(1):e0190153. doi: 10.1371/journal.pone.0190153 (PMC5764252; doi:10.1371/journal.pone.0190153)
Supplement: S1 Table — List of specimens examined available in the Laboratório de Herpetologia da Universidade Federal Rural do Rio de Janeiro and in the Coleção de Herpetologia do Museu Nacional do Rio de Janeiro. (DOCX) [file pone.0190153.s001.docx]

*Thoropa miliaris* (n = 111): BRAZIL: **Rio de Janeiro:** Angra dos Reis: Ilha Grande, RU9557

RU9551, RU9564, MNRJ45487, CAB1850, RU9560, MNRJ45530, MNRJ45486, MNRJ19940, MNRJ45519, MNRJ45491, MNRJ45490, MNRJ45499, MNRJ45529, MNRJ45527, MNRJ45480, RU6986, RU5432, RU6989, MNRJ48450, MNRJ45481, RU6988, MNRJ45489, MNRJ45520, MNRJ23624, MNRJ45525; Mangaratiba: Ilha da Marambaia, RU09139, RU9002, RU9285, RU09137, RU9284, RU8987, RU9282, RU74, RU64, RU1320, MNRJ9350, RU9003, RU261, RU73, MNRJ20148, MNRJ20081, RU9281, MNRJ20043, RU8926, RU8992, RU9274, RU260, RU8924, RU8925, MNRJ19964, RU137, RU135, MNRJ20045, RU1738, RU9000, RU9001, MNRJ20046; Ilha de Itacuruçá, RU9533, RU9371, RU9434, RU9517, RU9532, RU9514, RU9513, RU9519, RU9437, RU9436, RU9505, RU8974, RU9520, RU8976, RU9374, RU4865, RU9372, RU9103, RU8975; Duque de Caxias, MNRJ87490; Mangaratiba, RU5423, RU1490, RU1480; Itaguaí: RU9414, RU9415, RU6468, RU8006, RU8007, RU9422; Nova Iguaçú: MNRJ77400; Rio de Janeiro: MNRJ23111, MNRJ38847, MNRJ24976, RU9361, RU9362, RU9341, MNRJ43617, MNRJ38839, MNRJ24978, MNRJ24976, RU9343, MNRJ38840, RU9359, RU9357, MNRJ24979, MNRJ35453, MNRJ23219, MNRJ47507, MNRJ26493, MNRJ47898, MNRJ74077, MNRJ27530, MNRJ26493.

*Adenomera marmorata* (n = 185): BRAZIL: **Rio de Janeiro:** Angra dos Reis: Ilha Grande, MNRJ45791, MNRJ55735, MNRJ39338, MNRJ42380, MNRJ42363, MNRJ2187, MNRJ66242, MNRJ42381, MNRJ42372, MNRJ42364, MNRJ42379, MNRJ42378, MNRJ42373, MNRJ50225, MNRJ42366, MNRJ42367, MNRJ42371, MNRJ42365, MNRJ42377, MNRJ42375, MNRJ51865, MNRJ42389, MNRJ42368, MNRJ42374, MNRJ42370, MNRJ42376, MNRJ86345, MNRJ44626; Mangaratiba: RU1533, RU6179, RU1650, RU1651, RU4830, RU1551, RU3920, RU3922, RU1506, RU1452, RU1622, RU1510, RU1428, RU1587, RU1427, RU3917, RU1631, RU1595, RU1457, RU1507, RU1585, RU3915, RU1654, RU1548, RU1456, RU1588, RU1487,RU3921, RU3916, RU1549, RU1454, RU1530, RU1534, RU1508, RU1547, RU1532, RU3919, RU3918, RU1509, RU5605, RU1471, RU1653, RU1550, RU1485, RU1455; Ilha da Marambaia, RU9058, RU9059, RU9060, RU9072, RU9063, RU9071, RU9062, RU9069, RU9332, RU9070, RU9061, RU5407, RU9066, RU9067, RU9068, RU9065, RU5019, RU102, RU099, RU259, RU9064, RU385, RU255, RU097, RU8946; Ilha de Itacuruçá, RU8985, RU6026, RU6035, RU6025, RU6028, RU6032, RU9110, RU6024, RU5961, RU6290, RU6034, RU6215, RU6031, RU6029, RU5974, RU6027, RU6030, RU3579, RU5960; Ilha de Jaguanum, RU3363, RU3354, RU3321, RU3357, RU3353, RU3355, RU3362, RU3358, RU3356, RU3364, RU3320, RU3326, RU3313, RU3372; Duque de Caxias: MNRJ73938, MNRJ73937, MNRJ73939, MNRJ71610, MNRJ71611, MNRJ71612, MNRJ73395, MNRJ73936; Itaguaí: RU8536, RU6264, RU6270, RU6268, RU6258, RU6267, RU6259, RU5962, RU8537, RU6232; Paraty: RU8019, RU8022, RU8017, RU8025, RU8018, RU8024, RU8028; Rio de Janeiro: MNRJ64782, MNRJ64772, MNRJ64738, MNRJ64739, MNRJ13284, MNRJ64771, MNRJ27653, MNRJ13283, MNRJ65495, MNRJ64760, MNRJ3078.

*Boana albomarginata* (n = 201): BRAZIL: **Rio de Janeiro:** Angra dos Reis: RU6197, RU6196, RU6198; Ilha de Itanhangá, RU7572, RU7573, RU7577, RU7565, RU7570, RU7569, RU7576, RU7568, RU7567, RU7574, RU7578, RU7575, RU7563, RU7571; Ilha da Gipóia, RU7530, RU7539, RU7536, RU7532, RU7549, RU7535, RU7531, RU7550, RU7534, RU7529, RU7533, RU7551; Cacaria: RU7839, RU7838, RU7836, RU7841, RU7837, RU7840, RU7843, RU7835, RU7842; Itaguaí: MNRJ65547, MNRJ65551, MNRJ65550, MNRJ65549, MNRJ65554, MNRJ65553, MNRJ65556, MNRJ65552, MNRJ65555, MNRJ65548; Mangaratiba: Ilha da Marambaia, MNRJ20019, MNRJ20018, MNRJ20017, MNRJ81900, MNRJ81899, MNRJ20055, MNRJ20032, MNRJ47551, MNRJ20033, MNRJ20016, RU170, RU178, RU174, RU190, RU177, RU171, RU172, RU101, MNRJ19382, RU182, RU118, RU189, RU181, RU187, RU173, RU176, RU472, RU175, RU188, RU5286, RU8995, RU9073, RU8927; Ilha de Itacuruçá, RU09501, RU09498, RU09497, RU09499, RU9440, RU09500, RU09504, RU9441, RU09494, RU9439, RU4962, RU4963, RU4966, RU9370, RU4965, RU09490, RU09488, RU4961, RU09496, RU4964, RU9365, RU9369, RU09503, RU9408, RU09495, RU9368, RU09493, RU09486, RU9367, RU09492, RU9364, RU9366, RU09489, RU09487; Nova Iguaçú: MNRJ85893, MNRJ85892; Rio de Janeiro: MNRJ42981, MNRJ42980, MNRJ7658, MNRJ7655, MNRJ11316, MNRJ16740, MNRJ42921, MNRJ42976, MNRJ84986, MNRJ42975, MNRJ42979, MNRJ84987, MNRJ27696, MNRJ11313, MNRJ7657, MNRJ7656, MNRJ2462, MNRJ11315, MNRJ7653, MNRJ7652, MNRJ42925, MNRJ11317, MNRJ86156, MNRJ42922, MNRJ84989, MNRJ84985, MNRJ48023, MNRJ42978, MNRJ7654, MNRJ86155, MNRJ48024, MNRJ42977, MNRJ11314, MNRJ48022, MNRJ84988, MNRJ1378, MNRJ86157; Seropédica: MNRJ81590, MNRJ81589, MNRJ74952, MNRJ16853, MNRJ81587, MNRJ16880.

*Ololygon trapicheiroi* (n = 203): BRAZIL: **Rio de Janeiro:** Angra dos Reis: Ilha Grande, RU7015, RU7014, RU7016, RU6208, MNRJ64158, MNRJ48117, MNRJ86351, MNRJ86351, MNRJ48117, MNRJ64158, MNRJ39203, MNRJ39202, RU9608, RU9611, RU9598, RU9602, RU9620, RU9603, RU9612, RU9613, RU9616, RU9609, RU9618, RU9607, RU9597, RU9617, RU9619, RU9600, RU9606, RU9599, RU9604, RU9601, RU9610, RU9614, RU9605, RU9615; Ilha da Marambaia, RU8938, RU8939, RU8940, RU8941, RU9009, RU9096, RU9089, RU9092, RU9090, RU9095, RU9091, RU9093, RU9094, RU54, RU52, RU149, RU126, RU5278, RU57, RU66, RU55, RU53, RU6944, RU1722, RU4295, RU1725, RU266, RU151, RU75, RU5277, RU185, RU56, RU47, RU3650, RU150, RU1720, RU1724, RU124, RU127, RU186, MNRJ20089, MNRJ20092, MNRJ20091, MNRJ20090; Ilha de Itacuruçá, RU8978, RU4981, RU3573, RU3574, RU4979, RU3572, RU5966, RU6023, RU4977, RU3575, RU4980, RU4974, RU4976, RU4975, RU4982; Nova Iguaçú: MNRJ59842, MNRJ59839, MNRJ59841, MNRJ59832, MNRJ59837, MNRJ59833, MNRJ59834, MNRJ59835, MNRJ59840, MNRJ59839, MNRJ59832, MNRJ59842, MNRJ59841, MNRJ59833, MNRJ59834, MNRJ59837, MNRJ59840, MNRJ59835, MNRJ86494, MNRJ86495; Rio de Janeiro: MNRJ78382, MNRJ78381, MNRJ78380, MNRJ77369, MNRJ77364, MNRJ77370, MNRJ77366, MNRJ16972, MNRJ16970, MNRJ16968, MNRJ16967, MNRJ16969, MNRJ16971, MNRJ77372, MNRJ77371, MNRJ86303, MNRJ35121, MNRJ74622, MNRJ16046, MNRJ16044, MNRJ16045, MNRJ16042, MNRJ16037, MNRJ16039, MNRJ16036, MNRJ16038, MNRJ16048, MNRJ16040, MNRJ16043, MNRJ16035, MNRJ16041, MNRJ27618, MNRJ27622, MNRJ27621, MNRJ27620, MNRJ27573, MNRJ85002, MNRJ85001, MNRJ85004, MNRJ27669, MNRJ27661, MNRJ27662, MNRJ27677, MNRJ27680, MNRJ27681, MNRJ27659, MNRJ27660, MNRJ27667, MNRJ27686, MNRJ27683, MNRJ27658, MNRJ27672, MNRJ27673, MNRJ27688, MNRJ27664, MNRJ27668, MNRJ27682, MNRJ27670, MNRJ27678, MNRJ27679, MNRJ27657, MNRJ27687, MNRJ27573, MNRJ76762, MNRJ35121, MNRJ16972, MNRJ16968, MNRJ16970, MNRJ16969, MNRJ16967, MNRJ16971, MNRJ39439, MNRJ39440, MNRJ39438, MNRJ39442, MNRJ74622, MNRJ78380, MNRJ78381, MNRJ78382, MNRJ77364, MNRJ77369, MNRJ77366, MNRJ77370, MNRJ86303, MNRJ77371, MNRJ77372
