# Supplementary material for: Frog size on continental islands of the coast of Rio de Janeiro and the generality of the Island Rule
Source: PLoS One. 2018 Jan 11;13(1):e0190153. doi: 10.1371/journal.pone.0190153 (PMC5764252; doi:10.1371/journal.pone.0190153)
Supplement: S2 Table — Loadings of the first five principal componentes of the PCA with proportion of variance (PoV—%) and cumulative proportion of variance (CpoV—%). (DOCX) [file pone.0190153.s002.docx]

| ***Thoropa miliaris*** | | | | | |
| --- | --- | --- | --- | --- | --- |
|  | **Comp.1** | **Comp.2** | **Comp.3** | **Comp.4** | **Comp.5** |
| **SVL** | -0.2718 | -0.1204 | -0.0549 | 0.0376 | 0.1582 |
| **HL** | -0.2413 | 0.2761 | 0.0629 | -0.2989 | 0.7233 |
| **HW** | -0.2928 | -0.0813 | -0.0972 | 0.0506 | 0.1266 |
| **IND** | -0.2653 | -0.0254 | -0.1232 | -0.2415 | 0.1401 |
| **IOD** | -0.2705 | 0.0396 | -0.2213 | 0.0734 | -0.0619 |
| **ESD** | -0.3406 | 0.2199 | 0.6209 | -0.2199 | -0.4839 |
| **END** | -0.3396 | 0.1215 | -0.0356 | -0.2887 | -0.1781 |
| **ED** | -0.1844 | 0.7120 | -0.1300 | 0.6310 | -0.0299 |
| **TD** | -0.2484 | 0.0260 | -0.6720 | -0.2785 | -0.3552 |
| **FTL** | -0.2745 | -0.1729 | 0.1113 | 0.1809 | 0.0970 |
| **TL** | -0.2697 | -0.4976 | -0.0309 | 0.4217 | -0.0577 |
| **LL** | -0.2803 | -0.2063 | 0.1279 | 0.1543 | 0.0588 |
| **FL** | -0.2913 | -0.0978 | 0.1858 | 0.0357 | 0.0564 |
| **PoV** | 79.9225 | 5.6287 | 3.2914 | 3.0059 | 2.1107 |
| **CPoV** | 79.9225 | 85.5513 | 88.8427 | 91.8486 | 93.9595 |

| ***Adenomera marmorata*** | | | | | |
| --- | --- | --- | --- | --- | --- |
|  | **Comp.1** | **Comp.2** | **Comp.3** | **Comp.4** | **Comp.5** |
| **SVL** | -0.1781 | -0.1485 | 0.0863 | -0.2329 | 0.0451 |
| **HL** | -0.2738 | -0.2796 | 0.0592 | 0.0270 | -0.1280 |
| **HW** | -0.2949 | -0.3018 | 0.0700 | 0.1431 | -0.1386 |
| **IND** | -0.3494 | -0.3712 | 0.1229 | 0.3807 | 0.1018 |
| **IOD** | -0.2912 | -0.3131 | 0.1196 | 0.0348 | -0.3289 |
| **ESD** | -0.3238 | -0.0711 | -0.3233 | -0.0754 | 0.5934 |
| **END** | -0.4172 | 0.3008 | -0.7254 | 0.1250 | -0.1343 |
| **ED** | -0.3066 | 0.3382 | 0.0782 | -0.3092 | -0.5804 |
| **TD** | -0.4251 | 0.5757 | 0.5521 | 0.1755 | 0.3046 |
| **FTL** | -0.1108 | -0.0891 | 0.1009 | -0.2283 | 0.0631 |
| **TL** | -0.1204 | -0.0928 | 0.0027 | -0.6119 | 0.1763 |
| **LL** | -0.0751 | -0.0741 | 0.0174 | -0.1273 | 0.0211 |
| **FL** | -0.1160 | -0.0977 | 0.0507 | -0.4335 | 0.0920 |
| **PoV** | 39.6216 | 24.321 | 8.9126 | 6.3371 | 5.1497 |
| **CPoV** | 39.6216 | 63.9427 | 72.8552 | 79.1924 | 84.3421 |

| ***Boana albomarginata*** | | | | | |
| --- | --- | --- | --- | --- | --- |
|  | **Comp.1** | **Comp.2** | **Comp.3** | **Comp.4** | **Comp.5** |
| **SVL** | -0.2619 | -0.0742 | -0.1273 | -0.1392 | 0.0488 |
| **HL** | -0.2420 | 0.2550 | -0.2486 | -0.1757 | -0.6144 |
| **HW** | -0.2182 | -0.0279 | -0.1024 | -0.1438 | 0.0522 |
| **IND** | -0.3010 | -0.4643 | -0.3747 | 0.6749 | -0.2613 |
| **IOD** | -0.2525 | -0.0391 | -0.0365 | -0.2320 | -0.1226 |
| **ESD** | -0.3295 | 0.4700 | 0.0992 | 0.1903 | 0.0989 |
| **END** | -0.4016 | 0.4935 | 0.1747 | 0.3746 | 0.2034 |
| **ED** | -0.2458 | 0.0473 | 0.1693 | -0.3177 | -0.4559 |
| **TD** | -0.3268 | -0.4404 | 0.7697 | 0.0211 | -0.0545 |
| **FTL** | -0.2654 | -0.1087 | -0.1602 | -0.1899 | 0.2460 |
| **TL** | -0.2364 | -0.1430 | -0.1885 | -0.2555 | 0.2418 |
| **LL** | -0.2233 | -0.1016 | -0.1395 | -0.1570 | 0.2286 |
| **FL** | -0.2399 | -0.0919 | -0.1664 | -0.1262 | 0.3177 |
| **PoV** | 53.8251 | 12.2213 | 9.6728 | 7.0597 | 5.4459 |
| **CPoV** | 53.8251 | 66.0465 | 75.7194 | 82.7799 | 88.2251 |

| ***Ololygon trapicheiroi*** | | | | | |
| --- | --- | --- | --- | --- | --- |
|  | **Comp.1** | **Comp.2** | **Comp.3** | **Comp.4** | **Comp.5** |
| **SVL** | -0.0878 | -0.0099 | -0.0797 | -0.0988 | -0.2015 |
| **HL** | -0.2927 | -0.1192 | -0.2958 | -0.0799 | 0.2293 |
| **HW** | -0.1227 | -0.0115 | -0.0790 | -0.0882 | -0.1548 |
| **IND** | -0.1393 | 0.2598 | -0.0308 | -0.8649 | 0.3276 |
| **IOD** | -0.1305 | -0.0028 | -0.1417 | -0.0666 | -0.1384 |
| **ESD** | -0.5412 | -0.1584 | 0.2634 | 0.0393 | 0.1887 |
| **END** | -0.5812 | -0.1971 | 0.4386 | 0.1020 | -0.0462 |
| **ED** | -0.3229 | 0.0151 | -0.7343 | 0.2988 | 0.2552 |
| **TD** | -0.2179 | 0.9217 | 0.1121 | 0.2451 | -0.0787 |
| **FTL** | -0.0834 | -0.0129 | -0.1696 | -0.1527 | -0.4401 |
| **TL** | -0.0813 | -0.0637 | -0.0840 | -0.1255 | -0.4106 |
| **LL** | -0.1100 | -0.0007 | -0.1356 | -0.1058 | -0.3076 |
| **FL** | -0.2168 | 0.0104 | -0.1053 | -0.1098 | -0.4364 |
| **PoV** | 40.9463 | 16.6381 | 11.7144 | 9.8124 | 5.7359 |
| **CPoV** | 40.9463 | 57.5845 | 69.2989 | 79.1113 | 84.8472 |
